# When We Don’t Have All the Answers: Long COVID and the Need for Humility in Medicine

**DOI:** 10.31486/toj.25.0074

**Published:** 2025

**Authors:** Liza Di Leo Thomas

**Affiliations:** Department of Emergency Medicine, Ochsner Clinic Foundation, New Orleans, LA and The University of Queensland Medical School, Ochsner Clinical School, New Orleans, LA

*In these days of aggressive self-assertion, when the stress of competition is so keen and the desire to make the most of oneself so universal, it may seem a little old-fashioned to preach the necessity of this virtue, but I insist for its own sake, and for the sake of what it brings, that a due humility should take the place of honour on the list.*

–*Sir William Osler, to medical students*

*Uncertainty is an uncomfortable position. But certainty is an absurd one.*

–*Voltaire*

In early 2020, the coronavirus disease 2019 (COVID-19) pandemic began. It was a very difficult time for clinicians. We were building the plane as we flew it. We shared anecdotes to determine the best way to manage the influx of patients, some of whom were very sick. The “right way” to manage these critically ill patients was always changing. It was a scary time for us and was even scarier for our patients.

At that time, according to the World Health Organization (WHO), patients with severe acute respiratory syndrome coronavirus 2 (SARS-CoV-2) were expected to recover relatively quickly. In fact, in his opening remarks at a press briefing held on February 24, 2020, the director general of the WHO stated, “…for people with mild disease, recovery time is about two weeks, while people with severe or critical disease recover within three to six weeks.”^1^ However, some patients discovered they were not recovering as predicted and looked for others who were suffering like they were. The term “Long COVID” was created online by patients and is most likely the first illness named by patients on Twitter.^2^ It wasn’t until February 2021, that Dr Anthony Fauci, director of the National Institute of Allergy and Infectious Diseases, gave Long COVID an official name: post-acute sequelae of SARS-CoV-2 infection, or PASC. Still, despite the official name from Dr Fauci and the allocation of a large sum of money by the National Institutes of Health (NIH) to research PASC, the term Long COVID was accepted and used by many public health bodies in the United States, such as the Centers for Disease Control and Prevention (CDC).^3^ Three years into the pandemic, the National Academies of Science, Engineering and Medicine (NASEM) decided it was important to develop a single definition of Long COVID to recognize it as a disease that can affect anyone, regardless of age, race, or sex.^4^ The 2024 NASEM basic definition is as follows: “Long COVID (LC) is an infection-associated chronic condition that occurs after SARS-CoV-2 infection and is present for at least three months as a continuous, relapsing and remitting, or progressive disease state that affects one or more organ systems.”^4^ The extended definition clearly states that Long COVID occurs after acute SARS-CoV-2 infection but does not require laboratory confirmation or other proof of initial infection. See Table 1 for the full definition.

**Table 1.** National Academies of Sciences, Engineering, and Medicine Long COVID Definition^4^

Long COVID (LC) is an infection-associated chronic condition that occurs after SARS-CoV-2 infection and is present for at least three months as a continuous, relapsing and remitting, or progressive disease state that affects one or more organ systems.
**LC manifests in multiple ways.** A complete enumeration of possible signs, symptoms, and diagnosable conditions of LC would have hundreds of entries. Any organ system can be involved, and LC patients can present with **single or multiple symptoms, such as** shortness of breath, cough, persistent fatigue, post-exertional malaise, difficulty concentrating, memory changes, recurring headache, lightheadedness, fast heart rate, sleep disturbance, problems with taste or smell, bloating, constipation, and diarrhea. **single or multiple diagnosable conditions**, **such as** interstitial lung disease and hypoxemia, cardiovascular disease and arrhythmias, cognitive impairment, mood disorders, anxiety, migraine, stroke, blood clots, chronic kidney disease, postural orthostatic tachycardia syndrome (POTS) and other forms of dysautonomia, myalgic encephalomyelitis/chronic fatigue syndrome (ME/CFS), mast cell activation syndrome (MCAS), fibromyalgia, connective tissue diseases, hyperlipidemia, diabetes, and autoimmune disorders such as lupus, rheumatoid arthritis, and Sjogren's syndrome.
**Important Features of Long COVID** LC can follow asymptomatic, mild, or severe SARS-CoV-2 infection. Previous infections may have been recognized or unrecognized. LC can be continuous from the time of acute SARS-CoV-2 infection or can be delayed in onset for weeks or months following what had appeared to be full recovery from acute infection. LC can range from mild to severe. It can resolve over a period of months or can persist for months or years. LC can exacerbate pre-existing health conditions or present as a new condition. LC can affect children and adults, regardless of health, disability, or socioeconomic status, age, sex, gender, sexual orientation, race, ethnicity, or geographic location. LC can impair individuals’ ability to work, attend school, take care of family, and care for themselves. It can have a profound emotional and physical impact on patients and their families and caregivers. LC can be diagnosed on clinical grounds. No biomarker currently available demonstrates conclusively the presence of LC.

SARS-CoV-2, severe acute respiratory syndrome coronavirus 2.

At the American Medical Association (AMA) House of Delegates Special Meeting in June 2021, the delegates recognized that Long COVID should be acknowledged as the significant crisis that it is. The delegates proposed “the development of an ICD-10 [International Classification of Diseases 10th revision] code or family of codes to recognize Post-Acute Sequelae of SARS-CoV-2 infection (‘PASC’ or ‘long COVID’) and other novel post-viral syndromes as a distinct diagnosis.”^5^ This proposal was made more than a year after patients first began to see they were not getting better. However, it was still a step in the right direction.

In March 2022, as part of the AMA series “What Doctors Wish Patients Knew,” Dr Devang Sanghavi, intensivist and medical director of the medical intensive care unit at Mayo Clinic in Jacksonville, Florida, identified 3 categories of Long COVID.^6^ Because acute SARS-CoV-2 infection causes cellular damage that can cause lingering symptoms, the first category is patients who do not recover fully because of direct cell damage from the virus. This type of Long COVID can occur in patients who were not hospitalized. The second category of Long COVID is patients who were admitted and were bedbound for a prolonged period of time. This chronic hospitalization can lead to muscle weakness and cognitive dysfunction similar to post-ICU care syndrome. The third category of Long COVID is patients who initially recovered but then developed symptoms.^6^

Of course, before diagnosing a patient with Long COVID, other conditions must be ruled out; it's important to determine if the patient's symptoms are attributable to a post-viral syndrome or some other new diagnosis.^6^

I write about this topic not only as a physician but also as a Long COVID patient myself. Two weeks after an acute infection with SARS-CoV-2 in December 2020, when the symptoms of the acute infection had just about resolved, I found myself with new, unexplained symptoms. I was lightheaded when I stood for any period of time. I had paresthesias in my hands and feet. The muscles in my legs were causing such pain I felt as if I had run a marathon. Walking was difficult; my legs were heavy, and I felt like I was walking through sand.

Previously, I had been healthy. As a mom of 5 and an emergency medicine physician, I was constantly busy with work and family but still found time to exercise regularly and socialize outside of work. Over the next several months, I was eventually diagnosed with small fiber neuropathy and dysautonomia. I was able to walk normally at that point, albeit more slowly than normal, but I still had fatigue and the severe pain in the muscles of my legs. I was no longer able to endure the physical demands of the emergency department. I felt socially isolated because I had lost my emergency room family and was too fatigued to socialize, especially in the evenings. My exercise classes were out of the question.

Before I received my diagnoses of small fiber neuropathy and dysautonomia, I saw many specialists and had a multitude of tests run. Each test came back normal. I felt frustrated: I had never felt worse. Why doesn’t it show up in tests? I began to doubt myself and doubt my illness—that is, until I’d have a bout of post-exertional malaise, feeling like I had the flu just because I had been upright for too long that day. I knew something was terribly wrong. My symptoms didn’t fit the textbook, and I worried that doctors would not believe that I was really sick. I wanted my old life back so badly. Fortunately, my primary care doctor and a few other specialists believed me. Although I was told to eat better and get out and exercise on more than one occasion, my primary care doctor was wonderful. She admitted her time limitations, so she asked me to continue my own research and bring her what I found. Through my research I discovered the benefits of low-dose naltrexone, a medication that I have been taking for more than a year now and that has slightly improved my fatigue. The naltrexone, along with salt, fluids, and pacing my activities, has allowed me to return to work in an administrative role and enjoy my family as my kids grow older. My life is nowhere near what it used to be, but my quality of life has improved tremendously from what it was in early 2021. I am reminded, however, that I am still sick when I push myself beyond my energy envelope. Then, the muscle pain returns with a vengeance, or the flu-like symptoms confine me to bed for a day or so to recover.

The pathophysiology of Long COVID is still being studied, but Long COVID research has lagged behind the study of acute SARS-CoV-2 infection.^7^ Research has been slow for several reasons. First, it took a while for the medical establishment to recognize that Long COVID was an important clinical entity, and some people in the medical establishment still don’t believe Long COVID is a real condition. Second, no confirmatory biomarker or clinical test for Long COVID has been found. Third, research into Long COVID initially was allocated few resources and little financial support. However, thanks to programs like the NIH program Researching COVID to Enhance Recovery (RECOVER), with $1.4 billion allotted, the pathophysiology and epidemiology of Long COVID can be studied in depth. In a 2024 review, Peluso and Deeks summarized the current research, showing what we know about Long COVID to date. The Figure summarizes the proposed biological mechanisms of Long COVID.^7^

**Figure.**
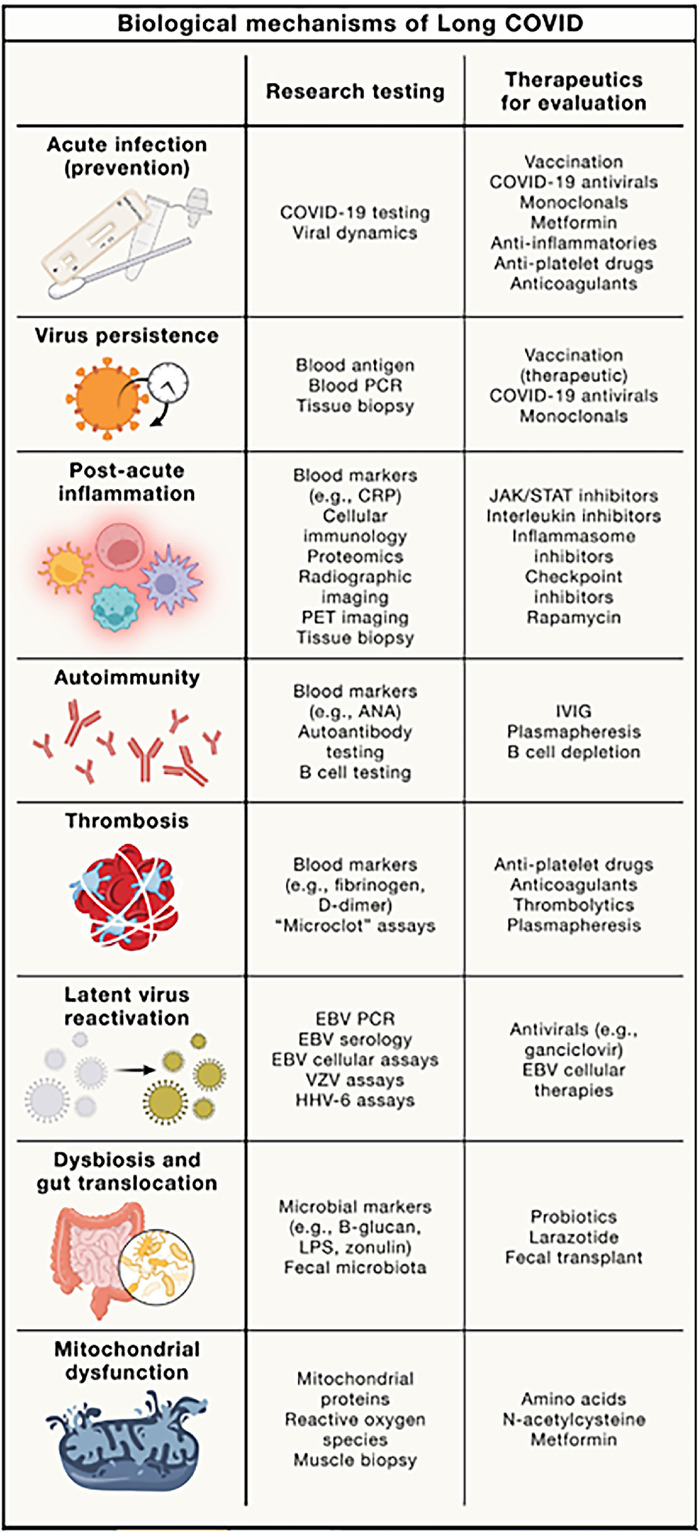
**Proposed biological mechanisms of Long COVID.** (Republished from Peluso and Deeks under the Creative Commons CC-BY license.^7^) ANA, antinuclear antibodies; CRP, C-reactive protein; EBV, Epstein-Barr virus; HHV-6, human herpesvirus 6; IVIG, intravenous immunoglobulin; JAK/STAT, Janus kinase/signal transducer and activator of transcription; LPS, lipopolysaccharide; PCR, polymerase chain reaction; PET, positron emission tomography; VZV, varicella-zoster virus.

Globally, the incidence of Long COVID continues to increase. In 2023, the cumulative global incidence of Long COVID was approximately 400 million individuals with an estimated “annual economic impact of approximately $1 trillion—equivalent to about 1% of the global economy.”^8^ According to the CDC, in 2022, 6.9% of adults and 1.3% of children (roughly 17 million and 1 million, respectively) in the United States reported ever experiencing Long COVID.^9^ While Long COVID can occur in anyone who contracts an acute SARS-CoV-2 infection, some groups are at higher risk of developing Long COVID: females, people with underlying conditions, people who experienced more severe outcomes of COVID-19, and people of Hispanic ethnicity.^9^ Of those who reported they had experienced Long COVID, approximately 1 in 5 adults reported significant limitations in their activities of daily living.^9^ In May 2023, these findings were corroborated. Robertson et al reported in *Clinical Infectious Diseases* that Long COVID affected 7.3% of US adults (approximately 18 million), and 25% of those affected felt their daily activities were limited secondary to the disease.^10^ The prevalence was higher among females, patients with comorbidities, patients who had not been boosted, and patients who had not been vaccinated.^10^

Symptoms of Long COVID can range from slightly disabling to being bedbound or unable to leave the house. The highest incidence of Long COVID occurs in patients infected with the early strains of the pandemic, and some evidence suggests that the risk of Long COVID is less with the Omicron and Delta variants.^11^ Whether reinfection with SARS-CoV-2 increases the risk of developing Long COVID is unknown, but a large study by Bowe et al suggests that it does.^12^ Further studies need to be done on the role of vaccination and antivirals in preventing Long COVID.

Also important to mention is the group of patients who developed a Long COVID–like condition after being vaccinated, known as post-vaccination syndrome. Researchers at Yale University are currently studying post-vaccination syndrome in the LISTEN (Listen to Immune, Symptom and Treatment Experiences Now) study.^13^

The exact number of patients with Long COVID or with post-vaccination syndrome cannot be truly known because many patients may be misdiagnosed, such as patients who actually have Long COVID but receive a new diagnosis attributed to a recent SARS-CoV-2 infection. Also, many patients may not be diagnosed at all if their physician does not recognize Long COVID.

Research must continue to find appropriate biomarkers, but until then, physicians must remain vigilant and confident in their clinical diagnostic capabilities. Currently, Long COVID is a clinical diagnosis. Listening to the patient is crucial.

In the NASEM definition, Long COVID is defined as an infection-associated chronic condition,^4^ a term used to describe any condition that begins after an infection and from which patients do not completely recover. In addition to Long COVID, myalgic encephalomyelitis/chronic fatigue syndrome, posttreatment Lyme disease syndrome, and multiple sclerosis after Epstein Barr are all types of infection-associated chronic conditions. Patients suffered with these conditions long before the start of the pandemic, but infection-associated chronic conditions have been underrecognized, with many patients feeling that their physicians do not believe them. There is a great deal of overlap in the symptoms of patients with Long COVID and patients with other infection-associated chronic conditions. According to the CDC, these symptoms can include tiredness or fatigue that interferes with daily life; flu-like symptoms including muscle pain, headache, sweating, irritability, and general feelings of sickness; symptoms that get worse after physical or mental effort (also known as post-exertional malaise); difficulty thinking or concentrating (sometimes referred to as brain fog) and trouble finding words; chronic or recurrent joint pain; and sleep problems.^14^ In June 2023, The National Academies Forum on Microbial Threats and Forum on Neuroscience and Nervous System Disorders hosted a workshop to discuss opportunities to advance research and treatment of infection-associated chronic illnesses.^15^ Because most medical schools do not teach about infection-associated chronic illnesses, patient groups and national organizations such as NASEM recognize that awareness needs to be increased. Fortunately, for patients who have been suffering from infection-associated chronic illnesses for years, the rise in Long COVID cases has brought about greater awareness that chronic conditions can indeed develop after an infection. However, many patients experiencing these illnesses face barriers in seeking diagnoses, treatment, and care, and even in just being believed. The link between infection and lasting symptoms may be poorly understood, but the conditions can be serious, potentially triggering long-term problems in many organ systems.^16^

When there is a paucity of information available, who becomes the expert? The answer to this question is simple: the patient. The patient is living with the condition and experiencing the symptoms, and frequently, the patient's life has been greatly affected. In the hope of returning their lives to their pre-illness state, most patients research their own conditions. As physicians, we must accept that these patients may know more than we do about their condition. This idea can be difficult to accept because we are supposed to be the experts, the ones who can advise patients on the best tests and the best possible management of their conditions. Listening to our patients with Long COVID and with other infection-associated chronic conditions is critically important. When our patients arrive with an internet search of articles, our job, rather than providing a fix, becomes providing guidance. We have been trained to critically analyze and clinically apply research as appropriate. We know when listening to anecdotal evidence is helpful. Still, given our time constraints, physicians have tremendous difficulty keeping abreast of all the new information that is available. For our patients, however, taking care of a chronic illness can be a part-time or even full-time job. Most have done the research themselves. Let your patients be your partners. Our patients will look to us not just for answers but also for validation. Letting them know that we hear them, we believe them, and we can work with them to interpret the information available on the internet becomes much more important than finding a specific diagnosis or cure.

When we apply the diagnosis that makes the most sense based on our training, we may unintentionally leave patients feeling dismissed. It is more honest—and often more compassionate—to admit when we don’t have all the answers, while acknowledging that we believe them and are committed to working together to find answers. This approach is far more respectful than attributing symptoms we don’t fully understand to a psychiatric etiology. Too many Long COVID patients have presented to their physicians with symptoms such as post-exertional malaise and have been dismissed. Post-exertional malaise, which is a hallmark symptom of myalgic encephalomyelitis/chronic fatigue syndrome and a symptom for many patients with Long COVID, is indeed an unusual presentation when we try to reconcile the condition with what we know about normal human physiology. Patients with post-exertional malaise can develop flu-like symptoms, brain fog, or extreme fatigue after an activity that may seem minimal to others, and the malaise can develop anywhere from 12 to 48 hours after the activity.^17^ For most medical conditions, recommending exercise, sometimes graded exercise therapy, is appropriate to help the patient to improve and recover. However, for patients with post-exertional malaise, graded exercise therapy can be dangerous. Exercising outside of their energy envelope can cause a crash that can last hours to days to months. The mechanism of post-exertional malaise has been studied. In a 2024 study, healthy patients and patients with a diagnosis of myalgic encephalomyelitis/chronic fatigue syndrome were asked to perform cardiopulmonary stress tests 2 days in a row.^18^ The healthy controls had similar findings on both days, whereas the patients with myalgic encephalomyelitis/chronic fatigue syndrome had more severe impairment status on day 2. The findings were consistent with autonomic nervous system dysregulation of blood flow and oxygen delivery rather than deconditioning.^18^ In another study, blood and skeletal muscle biopsies were obtained before and after a maximal exercise test from patients with Long COVID and from controls who had completely recovered from a SARS-CoV-2 infection.^19^ Overall, the data suggested that the poor performance of patients with Long COVID is related to a higher proportion of quickly fatiguing muscle fibers (high-fatigable glycolytic fibers) and an impaired function of the mitochondria. The process by which mitochondria convert nutrients into energy appears to be disrupted in patients with Long COVID.^19^

Pacing activities should be recognized as a potential management tool for post-exertional malaise. Although a review of the literature about the benefit of pacing in patients with Long COVID is inconclusive, here is an instance when listening to our patients is of the utmost importance. Pacing was shown to work in patients with fatiguing illnesses prior to the pandemic.^20^ If patients report that they develop more symptoms or their symptoms worsen when they do more than their new body can tolerate, the recommendation should be to keep activity within the patient's energy envelope and to pace activities, whether physical, cognitive, or emotional. Pacing could lead to some improvement in patients’ fatigue and other symptoms and most likely will improve their quality of life.

I recognize that my experience as a patient with Long COVID is not the usual one. When I was terrified because of the new symptoms I was experiencing, I reached out to my colleagues for help. Most patients are not so fortunate, and many patients feel dismissed or not believed by their doctors, a term that has come to be known as *medical gaslighting*.

The term gaslighting has become prevalent in popular culture. In fact, the word was Merriam-Webster's word of the year in 2022.^21^ The term first originated from the 1938 play “Gas Light” (and later, the movie) that tells the story of a woman who was manipulated by her husband into believing she was going insane. One of the husband's acts was to cause the gas lanterns in the house to dim and to tell his wife he did not see it happen. Medical gaslighting has been defined as “an act that invalidates a patient's genuine clinical concern without proper medical evaluation, because of physician ignorance, implicit bias, or medical paternalism.”^22^ How can we be sure we are not gaslighting our patients? By remaining humble.

Dr Jack Coulehan, Professor Emeritus at Stony Brook University, describes humility in medicine as manifesting 3 qualities: “unflinching self-awareness; empathic openness to others; and a keen appreciation of, and gratitude for, the privilege of caring for sick persons.”^23^ When we are humble in our practice, we accept that there are things we do not know. Such acceptance can be difficult because of the enormous responsibility we have as doctors to care for our patients and the pressure we place upon ourselves to make our patients better. But it is important to recognize that being humble is not the same thing as being mediocre or indecisive, just as being uncertain does not mean being incompetent. In their article, rheumatologists Kelly and Panush state, “Diagnostic and epistemologic humility should be a more common default position than is usually taken. We need to be comfortable with the uncomfortable. It better serves our science and art, and our patients. While uncertainty can be distressing, the alternative is indeed more perilous.”^24^ When we are humble with our patients, we are better communicators. When we are better communicators, our patients have better health outcomes.^25^

The way we communicate with our patients, especially those with invisible illnesses such as Long COVID, becomes extremely important in establishing trust. *Never-words* are words or phrases that when used with our patients not only “lack benefit but can also cause emotional harm and accentuate power differences….”^26^ For example, rather than telling a patient, “Everything will be fine,” instead say something like, “I’m here to support you throughout this process.” This choice of words is more realistic and less dismissive. Instead of using the words “fight” or “battle,” say, “We will face this difficult disease together.” This kind of language avoids implying that the patient's strong will alone can overcome the condition, which could leave the patient feeling guilty if they don’t get better. Although these never-words were described in the context of communicating with patients who are seriously ill, some of these suggestions can be extrapolated to all patient populations. In Table 2, I have provided my own never-words, based on my personal experience as a Long COVID patient. Fortunately, I am not the first author to suggest never-words for illnesses such as Long COVID, myalgic encephalomyelitis/chronic fatigue syndrome, and other complex chronic illnesses.^26,27^ The fact that the Lee et al^26^ and the Smyth and Blitshteyn^27^ papers on never-words were only recently published (2024 and 2025, respectively) speaks to the increasing awareness of the importance of communication with this patient population. However, we still have a long way to go.

**Table 2.** Never-Words for Patients With Invisible Illnesses – Liza Di Leo Thomas, MD

Never-Word Phrase	Alternative	Explanation
“But you look fine.” “At least you don’t look sick.”	“It must be frustrating to feel so awful.”	Telling patients they look fine may make them feel like you are doubting their illness, even if you are not.
“Great news! All your tests are negative!”	“The good news is we have ruled out (A, B, C) by doing these tests. But I believe there is something wrong, and I will work with you to figure it out.”	Some patients may not feel like it is great news that test after test is negative when they know something is absolutely wrong with them.
“You can’t believe everything you see online. Let's not diagnose you based on a TikTok influencer.”	“Thank you for taking such an interest in your illness and advocating for yourself. Let's talk about what you saw online.”	Online communities have become a huge source of support for patients with Long COVID and other invisible illnesses. Hearing someone else describe the same symptoms can be incredibly validating. Avoid dismissing these sources outright.
“At least you’re not bedbound, wheelchair bound, don’t have cancer, et cetera, et cetera, et cetera….”	“It sounds like you are suffering with this quite a bit. Can you explain how it has affected your quality of life?”	Avoid starting with “at least,” which can sound dismissive or invalidating. Instead, explore how the illness impacts the patient's life.
“We don’t know much about this illness. There's nothing I can do for you.”	“I realize the research on Long COVID is slow to come, and I can’t imagine how frustrating that is for you. Let's see what we can do right now to treat some of your symptoms and improve your quality of life.”	Even without definitive treatments, providers can offer symptom management and hope. Acknowledging the patient's frustration while focusing on what can be done is empowering.

As we face another summer surge of COVID, recognizing and validating the experiences of patients who don’t recover are essential. Many of these individuals may have previously encountered doubt or dismissal from other physicians, making it harder for them to trust us. The key is humility. Even when we don’t have all the answers, our patients need to know we believe their symptoms are real. Practicing medicine with humility is one of the most powerful ways we can build that trust.

## References

[1] WHO Director-General's opening remarks at the media briefing on COVID-19 - 24 February 2020. World Health Organization. February 23, 2020. Accessed July 31, 2025. who.int/director-general/speeches/detail/who-director-general-s-opening-remarks-at-the-media-briefing-on-covid-19—24-february-2020

[2] Callard F, Perego E. How and why patients made Long Covid. Soc Sci Med. 2021;268:113426. doi: 10.1016/j.socscimed.2020.113426 33199035 PMC7539940

[3] Long COVID or post-COVID conditions. U.S. Centers for Disease Control and Prevention. Updated December 16, 2022. Accessed July 31, 2025. archive.cdc.gov/www_cdc_gov/coronavirus/2019-ncov/long-term-effects/index.html

[4] National Academies of Sciences, Engineering, and Medicine. A Long COVID Definition: A Chronic, Systemic Disease State with Profound Consequences. The National Academies Press; 2024. doi: 10.17226/27768 39110819

[5] Berg S. More resources needed to help millions living with “long COVID.” American Medical Association. Published June 16, 2021. Accessed July 31, 2025. ama-assn.org/delivering-care/public-health/more-resources-needed-help-millions-living-long-covid

[6] Berg S. What doctors wish patients knew about long COVID. American Medical Association. Published March 11, 2022. Accessed July 31, 2025. ama-assn.org/delivering-care/public-health/what-doctors-wish-patients-knew-about-long-covid

[7] Peluso MJ, Deeks SG. Mechanisms of long COVID and the path toward therapeutics. Cell. 2024;187(20):5500-5529. doi: 10.1016/j.cell.2024.07.054 39326415 PMC11455603

[8] Al-Aly Z, Davis H, McCorkell L, Long COVID science, research and policy. Nat Med. 2024;30(8):2148-2164. doi: 10.1038/s41591-024-03173-6 39122965

[9] CDC science and the public health approach to Long COVID. U.S. Centers for Disease Control and Prevention. Published July 24, 2025. Accessed July 31, 2025. cdc.gov/covid/php/long-covid/index.html#cdc_generic_section_8-explore-cdc-data

[10] Robertson MM, Qasmieh SA, Kulkarni SG, The epidemiology of long coronavirus disease in US adults. Clin Infect Dis. 2023;76(9):1636-1645. doi: 10.1093/cid/ciac961 36542514

[11] Morioka S, Tsuzuki S, Suzuki M, Post COVID-19 condition of the Omicron variant of SARS-CoV-2. J Infect Chemother. 2022;28(11):1546-1551. doi: 10.1016/j.jiac.2022.08.007 35963600 PMC9365517

[12] Bowe B, Xie Y, Al-Aly Z. Acute and postacute sequelae associated with SARS-CoV-2 reinfection. Nat Med. 2022;28(11):2398-2405. doi: 10.1038/s41591-022-02051-3 36357676 PMC9671810

[13] Bhattacharjee B, Lu P, Monteiro VS, Immunological and antigenic signatures associated with chronic illnesses after COVID-19 vaccination. medRxiv. Preprint posted online February 20, 2025. doi: 10.1101/2025.02.18.25322379

[14] About chronic symptoms following infections. U.S. Centers for Disease Control and Prevention. May 15, 2024. Accessed July 31, 2025. cdc.gov/chronic-symptoms-following-infections/about/

[15] National Academies of Sciences, Engineering, and Medicine. Toward a Common Research Agenda in Infection-Associated Chronic Illnesses: Proceedings of a Workshop. The National Academies Press; 2024. doi: 10.17226/27462 38648305

[16] Infection-associated chronic illnesses: data-driven solutions for diagnosis, treatment, and care. U.S. Department of Health and Human Services. Accessed July 31, 2025. healthdata.gov/stories/s/Infection-Associated-Chronic-Illnesses-2023-/giix-q93k/

[17] Myalgic encephalomyelitis (or encephalopathy)/chronic fatigue syndrome: diagnosis and management. National Institute for Health and Care Excellence. Published October 29, 2021. Accessed July 31, 2025. nice.org.uk/guidance/ng206 35438859

[18] Keller B, Receno CN, Franconi CJ, Cardiopulmonary and metabolic responses during a 2-day CPET in myalgic encephalomyelitis/chronic fatigue syndrome: translating reduced oxygen consumption to impairment status to treatment considerations. J Transl Med. 2024;22(1):627. doi: 10.1186/s12967-024-05410-5 38965566 PMC11229500

[19] Appelman B, Charlton BT, Goulding RP, Muscle abnormalities worsen after post-exertional malaise in long COVID. Nat Commun. 2024;15(1):17. doi: 10.1038/s41467-023-44432-3 38177128 PMC10766651

[20] Abonie US, Sandercock GRH, Heesterbeek M, Hettinga FJ. Effects of activity pacing in patients with chronic conditions associated with fatigue complaints: a meta-analysis. Disabil Rehabil. 2020;42(5):613-622. doi: 10.1080/09638288.2018.1504994 30449204

[21] Word of the Year 2022. ‘Gaslighting,’ plus ‘sentient,’ ‘omicron,’ ‘queen consort,’ and other top lookups of 2022. Merriam-Webster. Accessed August 14, 2025. merriam-webster.com/wordplay/word-of-the-year-2022

[22] Ng IK, Tham SZ, Singh GD, Thong C, Teo DB. Medical gaslighting: a new colloquialism. Am J Med. 2024;137(10):920-922. doi: 10.1016/j.amjmed.2024.06.022 38936758

[23] Coulehan J. On humility. Ann Intern Med. 2010;153(3):200-201. doi: 10.7326/0003-4819-153-3-201008030-00011 20679563

[24] Kelly A, Panush RS. Diagnostic uncertainty and epistemologic humility. Clin Rheumatol. 2017;36(6):1211-1214. doi: 10.1007/s10067-017-3631-8 28432522

[25] Ruberton PM, Huynh HP, Miller TA, Kruse E, Chancellor J, Lyubomirsky S. The relationship between physician humility, physician-patient communication, and patient health. Patient Educ Couns. 2016;99(7):1138-1145. doi: 10.1016/j.pec.2016.01.012 26830544

[26] Lee Adawi Awdish R, Grafton G, Berry LL. Never-words: what not to say to patients with serious illness. Mayo Clin Proc. 2024;99(10):1553-1557. doi: 10.1016/j.mayocp.2024.05.011 39177542

[27] Smyth NJ, Blitshteyn S. Language matters: what not to say to patients with Long COVID, myalgic encephalomyelitis/chronic fatigue syndrome, and other complex chronic disorders. Int J Environ Res Public Health. 2025;22(2):275. doi: 10.3390/ijerph22020275 40003500 PMC11855516

